# Experimental and Numerical Study on the Influence of Stress Concentration on the Flexural Stability of an Aluminium Hollow Tube

**DOI:** 10.3390/ma16041492

**Published:** 2023-02-10

**Authors:** Ganesh Radhakrishnan, Daniel Breaz, Sami Sulaiman Al Khusaibi, Amjad Juma Al Subaihi, Al Azhar Zahir Al Ismaili, AlSalt Malik AlMaani, Kadhavoor R. Karthikeyan

**Affiliations:** 1 Mechanical and Industrial Section, Engineering Department, University of Technology and Applied Sciences, Nizwa P.O. Box 477, Oman; 2 Department of Mathematics, “1 Decembrie 1918” University of Alba Iulia, 510009 Alba Iulia, Romania; 3 Department of Applied Mathematics and Science, National University of Science and Technology, Muscat P.O. Box 620, Oman

**Keywords:** hollow tube, flexural strength, stress concentration, flexural stiffness, bending load

## Abstract

In recent times, particularly in applications used to build various structures for construction purposes or machines, solid sections have been gradually replaced by hollow sections due to their attractive features such as being light weight and having high specific strength. In the present investigation, an attempt was made to investigate, in detail, the flexural capability of aluminium hollow tubes (AHTs) with square cross-sections. The objective of the investigation was to study the influence of stress concentration on the flexural behaviour of the hollow tube. The stress concentration factor considered in this investigation was holes of various cross-sections and quantities. Three-point bending tests with concentrated loads were conducted on specimens of a hollow tube with different stress concentrations such as circular holes, multiple circular holes, square holes and perforations. The load was applied manually during the bending test with appropriate increments. The bending test was carried out on specimens with support spans of 110, 130, 170 and 200 mm. The output measures of the study were maximum bending load, deflection and flexural stiffness. The output measures were analysed in detail in order to recommend the type and nature of stress concentration in a hollow tube applied to structural applications to ensure the safest workability. The flexural stability of the tube was analysed by experimental and numerical procedures, and the results were validated using an analytical approach. It was found that the results of all the approaches complement each other with a low significance of error. AHTs with a circular hole, multiple circular holes and perforations were observed to have better flexural stability than other AHTs such as AHTs with square hole and plain AHTs.

## 1. Introduction

In recent times, most of the engineering applications in the automobile, aerospace, structural member, etc., industries have consistently replaced conventional bulk materials with high-performance and high specific-strength materials such as super alloys and composites and are slowly replacing the solid sections with hollow sections, which not only minimizes the amount of material used in construction but also increases their strength and stability by several times, meeting the desired requirements for which it is intended for. Conventional bulky materials such as steel and iron were replaced by aluminium alloys, magnesium alloys, stainless steel, composite materials, etc., which offer superior specific strength and are economical too. The structural members used in engineering applications are available in many sections, either in the solid or hollow types. Hollow sections have more specific strength than that solid sections. Hollow sections are lighter than solid sections and, therefore, mostly preferred in many structural applications. Square and circular hollow tubes have many applications in the area of multidirectional loading due to their uniform geometry along two or more cross-sectional axes. This, in turn, has uniform strength across the complete structure, which makes them good choices for many structural applications. In spite of heat treatment and super finishing of the materials used in the structural applications, certain machining operations such as drilling, threading, riveting, etc., are mandatory in the case of assembling the structures. These secondary machining operations include the concentration of heavy stress at localized spots around the discontinuities such as holes, notches, etc., that exist in the members for fasteners or any assembly work [1,2,3]. This type of discontinuity is very difficult to eliminate in the assembly and leads to the concentration of stress at localized spots called stress risers. Stress concentration plays a vital role in affecting the performance of the structure. Stress concentration is the acquisition of a large quantity of stress across a structural member in a particular location due to a sudden change in the geometry or some kind of interruption. Sharp corners, cracks, holes, notches, etc., increase localized stress at the specific location around the interruption, which may lead to failure of the member. The influence of stress concentration need not be the same for solid and hollow sections. The type of stress concentration in the structural member subjected to various loads such as axial, bending and shear, greatly influence the flexural performance [4]. This work was an attempt to investigate, in a detailed manner, the influence of stress concentration in hollow sections subjected to bending load. A significant amount of research has been performed in the area of structural analysis for various sections against various loads including bending; however, flexural stability analyses of hollow sections and comparisons of the results with analytical and numerical analyses to minimize error have not been explored in depth. 

A hollow section used for a structure is a type of construction used in many structural applications. It can be circular, square, rectangular, or any other section based on the need. Square and circular hollow tubes have many applications in the area of multidirectional loading due to the uniform geometry along two or more cross-sectional axes. They have uniform strength across the complete structure, which makes them good choices for many structures [5,6,7,8]. They also have high resistance to torsion. Zingaila et al. [2] analysed the flexural behaviour of concrete UHPFRC/RC composite members and found that composite beams have enhanced flexural capacity, reduced crack dimensions and increased stiffness compared to that of RC beams. The three-point bending strength of thin silicon dies was evaluated by Tsai et al. [3] for its non-linearity behaviour during bending and concluded, finally, that the correction factor considered in the non-linearity theory highly depends on the deflection, span length, elastic modulus and thickness of the specimens. Wang et al. [5] analysed the tensile and compressive behaviour of carbon fibre-reinforced polyphenylenesulfide composites and found that the strength of the composite material was influenced more by the temperature than the stiffness of the fibre content in the composite. The failure of the composite material was due to the temperature gradient, which was evident from the SEM micrographs. The macro-fracture morphologies from the SEM micrographs illustrated the multiple failure modes due to thermal stress induced in the composite material. Lin et al. [7] compared the flexural behaviour of concrete-filled steel tubular frames with conventional reinforcement concrete structures and analysed various factors that influence the flexural behaviour of these structures. It was concluded in such a way that certain design modifications could be incorporated for the reasonable failure of steel-reinforced concrete members. Tuan et al. [9] have conducted the three-point bending test on concrete-filled steel tubes and observed that the buckling across the tube was delayed due to concrete. In addition, the concrete and steel in the structure complemented each other; therefore, the ductility of concrete was improved by steel, and the compression behaviour of steel was improved by concrete. Aydna et al. [10] investigated the bending and shear performance of the composite fabricated by pouring the waste polymer into the cold-formed I and U profile melds after homogenous pulping. The improved adherence between the steel and polypropylene increased the shear and bending capability. Changing the cross-sectional area in I and U beams under bending influenced the load at yielding, ductility and energy dissipation capacity. The addition of CFRP in I beams significantly increased the bending resistance in the free end region under the shear force. The addition of GFRP bars with better flexibility in I and U beams caused more ductile behaviour than CFRP bars. Zahedi et al. [11] performed their investigation on the flexural behaviour of steel tubes wrapped with carbon-reinforced polymer. Dimensional error was analysed during the study and found that the strength and stiffness were appreciably increased by using FRP laminates around the steel tube. Many research findings are available in this field of hollow tubes, with or without filling, and a considerable amount of scientific concepts and theories have been offered. Some of the important findings from the literature review include that the flexural behaviour of a hollow tube was greatly influenced by the cross-section of the tube, flexural strength of hollow tubes was improved by replacing conventional materials with composite tubes, numerical analysis using ANSYS for flexural testing helps to predict the performance accurately, and the flexural behaviour of a hollow tube using numerical analysis was compared with experimental testing and found to complement each other. The details of the experimentation of the present study are explained in the next section [9,12,13,14,15,16].

## 2. Experimentation

The flexural behaviour of hollow tubes was investigated. The material used in the investigation was commercially available aluminium hollow tubes of square cross-section with dimensions of 20 × 20 × 1.5. The aluminium used in the hollow tube was Al 6061. The composition and properties of AA 6061 are shown in Table 1 and Table 2, respectively.

**Figure 1:**
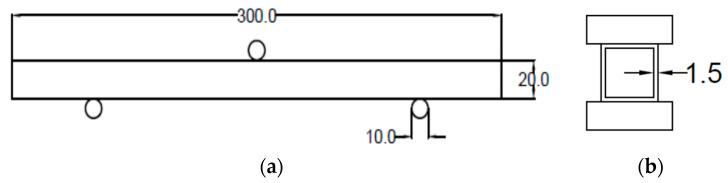
(**a**) Schematic diagram of flexural setup. (**b**) Cross section of AHT.

**Figure 2:**
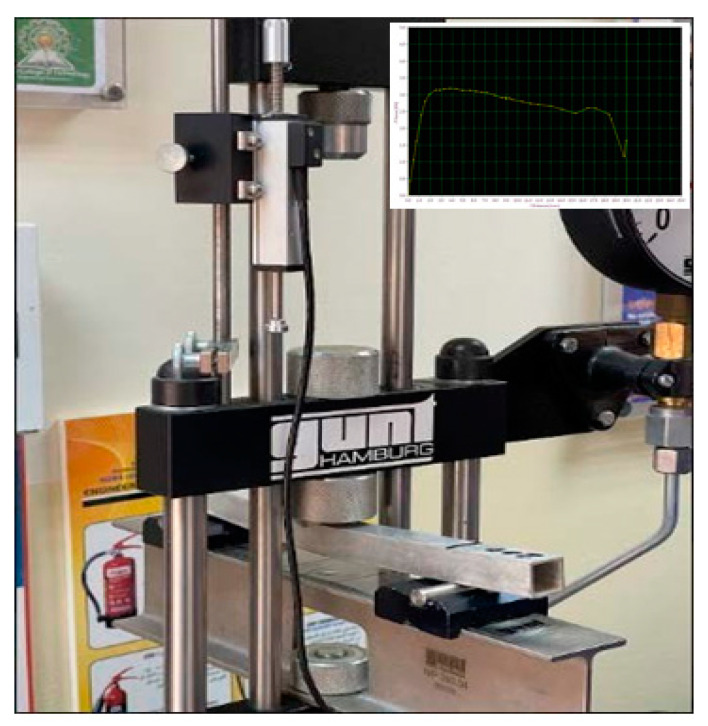
Photograph of experimental setup with a sample output.

The flexural capability of the aluminium hollow tubes (AHTs) was investigated in detail by conducting three-point flexural tests in a Universal tester machine of 20 kN capacity (Supplier: Gunt, Hamburg, Germany). A concentrated load was applied at the mid span of the specimen, perpendicular to the axis of the tube. The analysis of flexural capability of AHTs was carried out as a plain specimen and specimen with stress risers as well. Holes of different shapes and types were introduced to the AHTs in order to investigate their influence on the flexural capability of the specimen. Five different types of specimens were used in this flexural study. Plain AHTs, AHTs with a through square hole at the midspan on one of the lateral faces with dimension 10 mm, AHTs with through circular hole at the midspan on one of the lateral faces with dimension 5 mm, AHTs with multiple circular through holes at the midspan equally spaced on one of the lateral faces with dimensions 10 mm and 5 mm, and AHTs with perforations of multiple through holes of diameter 10 mm and 5 mm on adjacent lateral faces equally spaced throughout the length. Each test was repeated three times to obtain the results more accurately. The experiment was performed at normal room temperature, and the load applied during the test was manual. The output of the flexural test was taken through a data acquisition system (DAQ), attached to the machine and displayed. The performance measures considered in the flexural test were bending load, deflection and flexural stiffness. The critical bending load capacity and deflection were measured at the midspan of the specimen. The critical bending load was considered an indicator of the maximum resistance offered by the specimen against bending with minimum deflection. The flexural stiffness was calculated using the empirical relation between the critical bending load and the deflection. It was the measure of resistance against deformation, which was greatly influenced by the geometrical properties of the specimen and the loading characteristics. The schematic diagram of flexural test setup is shown in Figure 1a, and the cross-section of the specimen is shown in Figure 1b. The total length of the specimen was 300 mm, and the supports were placed at a distance of 15 mm from the end of the specimen on each side so that the effective length of the specimen under bending was 270 mm. The support rollers were 10 mm in diameter, and the contacts between the supports and the specimen were taken as point contacts with negligible friction. The thickness of all the specimens were 1.5 mm. The roller through which the bending load was applied was also of 10 mm in diameter, and the contact between the loading roller and the loading ram was also ignored during the experimentation. The experimental setup is shown in Figure 2. The same experimentation was also confirmed through a numerical approach using the ANSYS software package. The flexural test setup with the specimen was modelled using the SOLIDWORKS package and imported to ANSYS and analysed. Appropriate boundary conditions and constraints suitable for a three-point bending test were applied as per the standard procedure, and the analysis was carried out. Fine mesh with tetragonal elements of size 1 mm was employed in the finite element study. The load was treated as a concentrated type applied at the midspan of the beam in the transverse direction. The friction between the loading roller and the beam and between the supporting rollers and beam were ignored in the finite element study. This was due to the fact that the bending load was static. The load was applied on a regular and gradual increment basis of approximately 1 kN in each step. The test was repeated twice with the same load in order to ensure consistency of the results. The sensitivity of the element or mesh size was determined based on the literature and the dimensions of the AHT tube used in the study [17]. The material properties used in the experimental approach and the analytical approach were also used for the numerical analysis. Each and every specimen was tested numerically for different support spans under static flexural analysis [8,10,11,18,19,20]. The experimental bending load was applied to the specimen, and the maximum deflection was noted from the ANSYS results in order to confirm the accuracy of the test output. The model was created using a similar setup to the experimental approach. Bottom rollers were used as supports, whereas the top roller was used to apply the transverse load during the flexural test. The flexural performance of AHTs was validated using an analytical approach with the help of fundamental relations governing the flexural behaviour of a structure.

## 3. Results and Discussions

**Figure 3:**
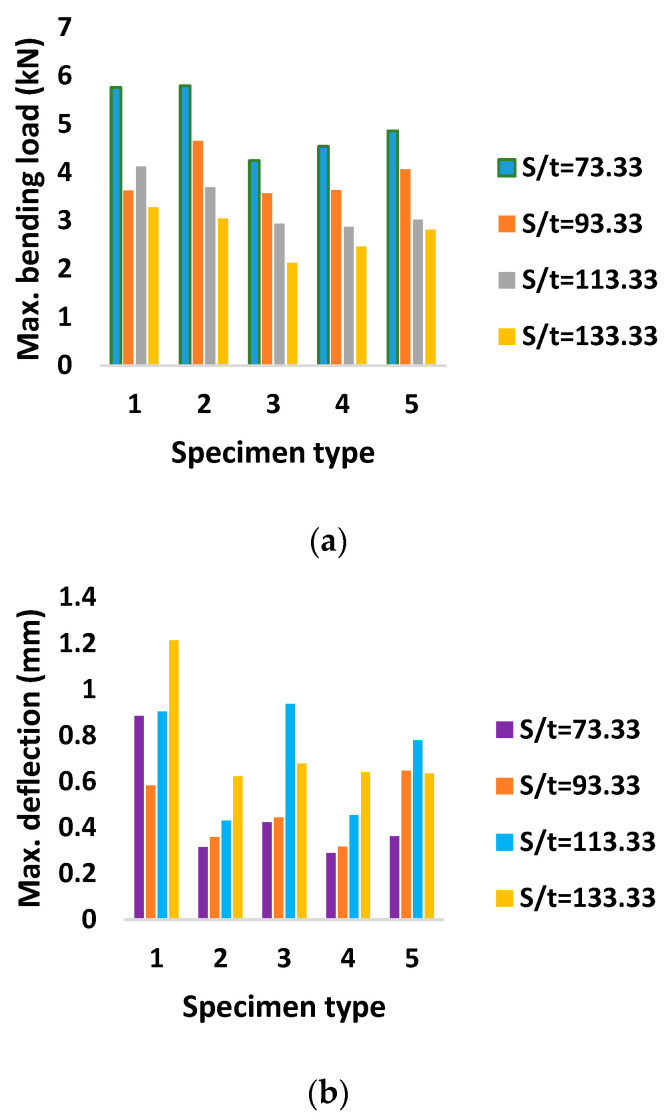

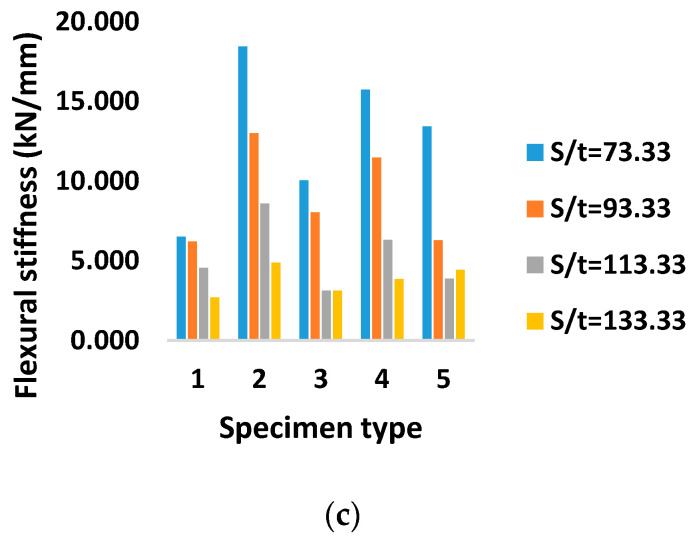
Experimental flexural performance measures (**a**) Maximum bending load (**b**) maximum deflection and (**c**) Flexural stiffness.

The flexural performance measures such as maximum bending load, maximum deflection and flexural stiffness, which were determined experimentally, are shown in Table 3. From the graphical illustrations shown in Figure 3a–c, it was observed that both the support span and type of discontinuity have significant effects on the flexural stability of AHTs. The support span aspect ratio S/t = 73.33 resulted in better flexural performance compared to that of other aspect ratios, irrespective of the type of discontinuity in the AHT. The lowest bending load capacity was observed for AHTs with square hole, which was attributed to the fact that the sharp corners of the square hole with stress concentration factors of 1.01 increased the concentration of stress around the discontinuity to the maximum level, which in turn increased the severity of failure and, therefore, reduced the bending load capacity. Maximum deflection was observed for the aspect ratio S/t = 133.33, and the least deflection was observed for the aspect ratio S/t = 73.33. The deflection measured at the centre of the specimen followed an increasing trend with respect to an increase in S/t. The longer the support span, the higher the deflection. A larger deflection of a member can result in permanent deflection, cracking and other damage. The larger deflection of one member in a structure may impact its integrity, that of any other member in the structure, or the stability of the entire structure as a whole. 

**Figure 4:**
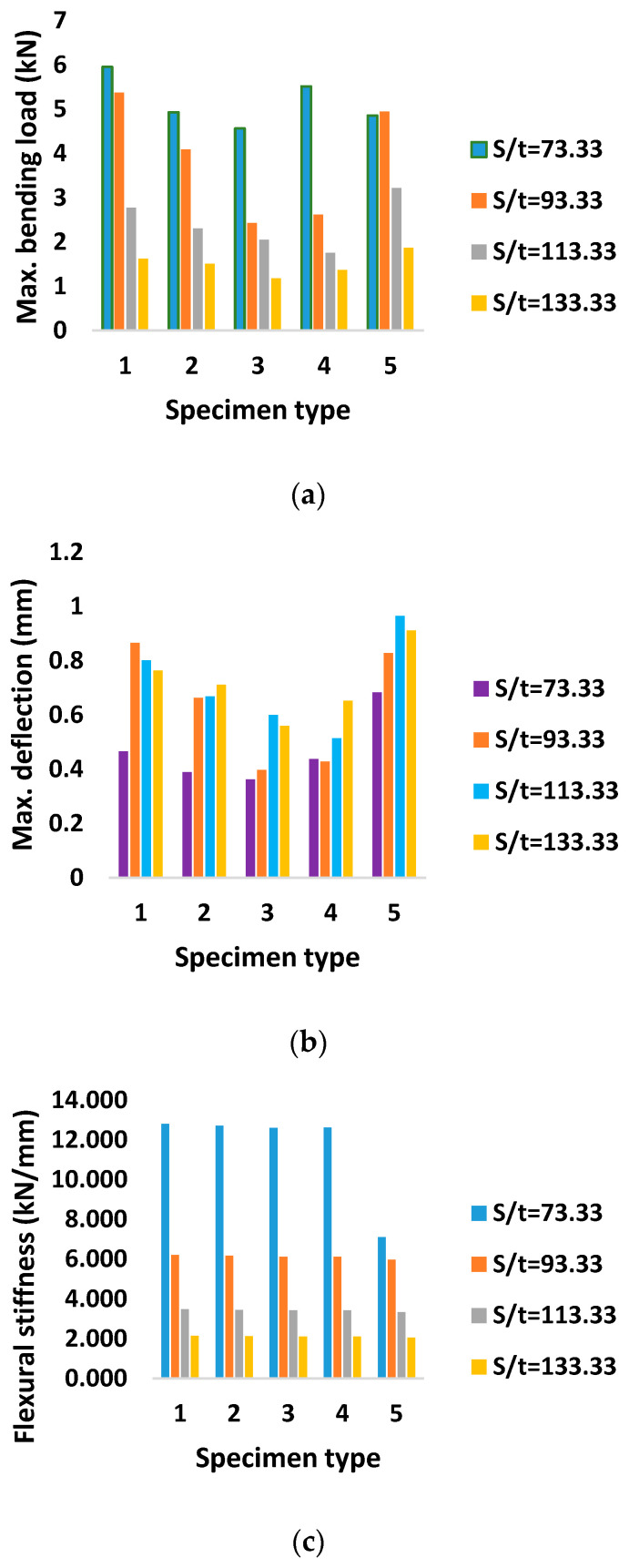
Numerical flexural performance measures (**a**) Maximum bending load (**b**) maximum deflection and (**c**) Flexural stiffness.

**Figure 5:**
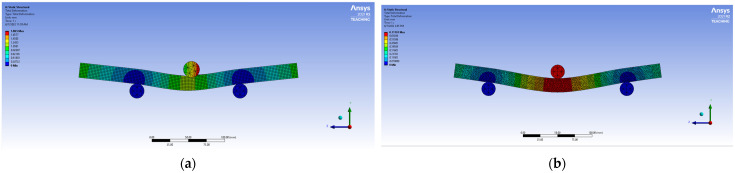

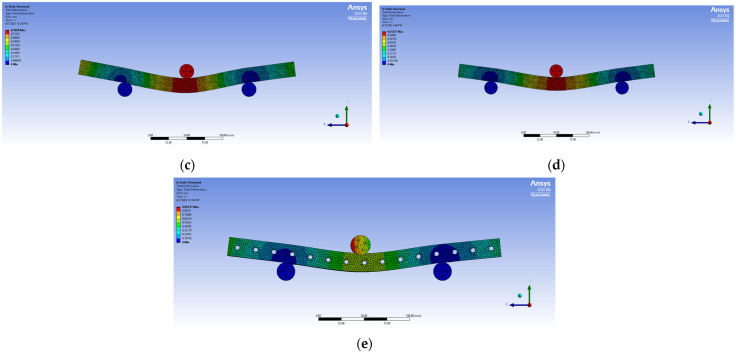
Sample outputs of numerical flexural performance at maximum deflection (**a**) plain AHT (**b**) AHT with circular hole (**c**) AHT with square hole (**d**) AHT with multiple circular holes and (**e**) AHT with perforations.

**Figure 6:**
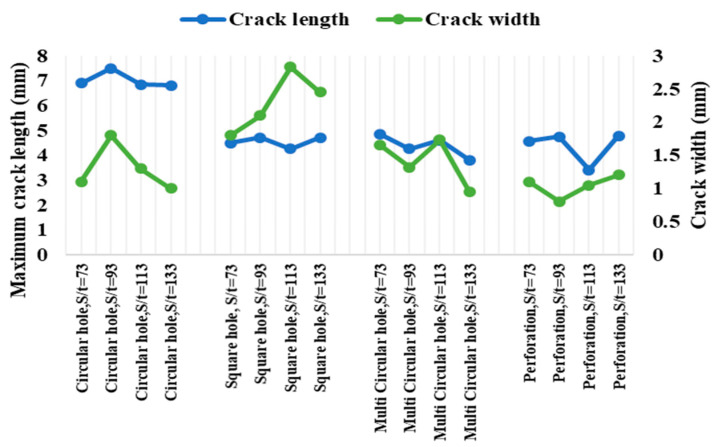
Crack dimensions of flexural failed specimens.

Flexural stiffness was observed to be maximum for AHTs with circular holes and perforations compared to that of AHTs with square holes and plain AHTs as well. This was due to the fact that creating additional holes on the specimen on either side reduced the effect of stress concentration, which in turn increased the stiffness of the specimen. The area moment of inertia of the specimen about minimum cross-sectional area was reduced for the specimens where the stress concentration effect was reduced by additional holes. This in turn increased the resistance to deformability or the flexural stiffness of the specimen under loading. The highest flexural stiffness in the range of 16 to 18 kN/mm was observed for AHTs with square holes. In order to ensure the consistency and accuracy of the results obtained through experimental approach, a numerical analysis using ANSYS software was made with constraints and boundary conditions similar to those of the conditions and assumptions made in the experimental approach. The results of the numerical study are listed in Table 4. The numerical outputs of the flexural test of AHTs are shown in Figure 4a–c. The outputs of the numerical study for deformed specimens with different types of discontinuities and support spans are shown in Figure 5a–e. It was noticed from the illustrations that the bending load capacity was maximum for the support span of S/t = 73.33 and the least for the support span of S/t = 133.33. The numerical results complemented the results obtained through the experimental approach. The influence of support span aspect ratio, S/t, was more on the flexural performance of AHTs rather than the type of discontinuity in the specimen. The numerical results of the flexural test of AHTs revealed that the consistency and accuracy of the experimentation were better and had the least difference in the results. The dimensions of cracks were measured for each failed specimen under flexural loading, tabulated in Table 5. The influence of the type of discontinuity and the support span aspect ratio, S/t, on the crack dimensions are shown in Figure 6. Crack dimensions were observed to be maximum for AHTs with circular and square holes compared to that of AHTs with multiple circular holes and perforations. The support span influenced the crack dimensions significantly. Crack propagation was high for AHTs with circular and square holes. This was due to the fact that the highest stress concentration initiates a crack at an earlier stage and propagates its growth significantly. The crack propagation seems to be faster for specimens with higher concentrations of stress risers. Average crack width seems to be minimum for AHTs with perforations, whereas they are maximum for AHTs with square holes. The lowest crack length of 3.4 mm was observed for AHTs with perforations specimen with aspect ratio, S/t = 113, whereas crack length was a maximum of about 7.5 mm for AHTs with circular holes at aspect ratio S/t = 93 [21,22].

## 4. Conclusions

An attempt was made successfully to study the flexural stability of aluminium hollow tube (AHTs) with different discontinuities in the form of holes of different geometries such as circular holes, square holes, multiple circular holes and perforations. The aspect ratio support span to thickness of the tube (S/t) was considered as the process parameter during the flexural test of AHTs. The study was performed in two aspects, experimental and numerical, and fruitful outcomes and conclusions were drawn from the study and listed below.

(i) The support span aspect ratio (S/t) significantly influenced the flexural behaviour of plain AHTs and AHTs with holes of different quantities and geometries.
(ii) Following the support span aspect ratio, the type of discontinuity in terms of shape, size and quantity significantly influenced the flexural stability of AHTs. This may be due to the fact that these discontinuities acted as stress risers and influenced the flexural capacity of the structure to a larger extent.
(iii) AHTs with a circular hole, multiple circular holes and perforations were observed to have better flexural stability than that of other AHTs such as AHTs with square holes and plain AHTs. This may be attributed to the fact that the sharp corners in the holes are the sources of crack initiation and propagation, which in turn lead to the failure of the specimen under loading.
(iv) Reducing the effect of stress concentration abruptly increased the flexural behaviour of AHTs by offering better flexural resistance.
(v) The results obtained through experimental and numerical approaches complemented each other with utmost accuracy and consistency. The assumptions and constraints made during the analysis were very close and complemented each other.

## Figures and Tables

**Table 1:** Chemical composition of AA6061.

Chemical Element	% Present
Manganese	0.0–0.15
Iron	0.0–0.7
Magnesium	0.80–1.2
Silicon	0.40–0.8
Copper	0.14–0.4
Zinc	0.0–0.25
Titanium	0.0–0.15
Chromium	0.04–0.35
Others	0.0–0.14
Aluminium	Balance

**Table 2:** Properties of AA6061.

Property	Value
Mass density	2.7 g/cm³
Melting Point	652 °C
Thermal expansion	23.4 × 10^−6^/K
Modulus of Elasticity	71 GPa
Thermal conductivity	168 W/mK
Electrical resistivity	0.042 × 10^−6^ Ωm
Proof stress	245 MPa
Tensile strength	255 MPa
Brinell hardness	94.5 HB

**Table 3:** Experimental flexural performance measures.

S. No.	Specimen	Stress Concentration Factor (K)	S/t	Maximum Bending Load (kN)	Maximum Deflection (mm)	Flexural Stiffness (kN/mm)
1	Plain	1	73.33	5.759	0.884	6.515
2			93.33	3.624	0.583	6.216
3			113.33	4.12	0.904	4.558
4			133.33	3.275	1.213	2.700
5	Circular hole	1.007	73.33	5.791	0.314	18.443
6			93.33	4.651	0.358	12.992
7			113.33	3.691	0.429	8.604
8			133.33	3.039	0.622	4.886
9	Square hole	1.01	73.33	4.243	0.423	10.031
10			93.33	3.568	0.444	8.036
11			113.33	2.936	0.936	3.137
12			133.33	2.123	0.677	3.136
13	Multiple circular holes	1.01	73.33	4.535	0.288	15.747
14			93.33	3.634	0.317	11.464
15			113.33	2.867	0.454	6.315
16			133.33	2.464	0.639	3.856
17	Perforation	1.014	73.33	4.856	0.362	13.414
18			93.33	4.063	0.647	6.280
19			113.33	3.016	0.779	3.872
20			133.33	2.808	0.634	4.429

**Table 4:** Numerical flexural performance measures.

S. No.	Specimen	Stress Concentration Factor (K)	S/t	Maximum Bending Load (kN)	Maximum Deflection (mm)	Flexural Stiffness (kN/mm)
1	AHT with circular hole	1	73.33	5.953	0.465	12.802
2			93.33	5.375	0.865	6.214
3			113.33	2.774	0.8	3.468
4			133.33	1.625	0.763	2.130
5	AHT with square hole	1.007	73.33	4.931	0.388	12.709
6			93.33	4.087	0.663	6.164
7			113.33	2.3	0.668	3.443
8			133.33	1.503	0.711	2.114
9	AHT with multiple circular holes	1.01	73.33	4.566	0.362	12.613
10			93.33	2.429	0.397	6.118
11			113.33	2.047	0.599	3.417
12			133.33	1.173	0.559	2.098
13	AHT with perforation	1.01	73.33	5.513	0.437	12.616
14			93.33	2.613	0.427	6.119
15			113.33	1.756	0.514	3.416
16			133.33	1.368	0.652	2.098
17	AHT with circular hole	1.014	73.33	4.855	0.683	7.108
18			93.33	4.942	0.828	5.969
19			113.33	3.216	0.965	3.333
20			133.33	1.865	0.911	2.047

**Table 5:** Crack dimensions of flexural failed specimens.

S. No.	Specimen	Aspect Ratio (S/t)	Stress Concentration Factor (K)	Crack Width (mm)	Crack Length (mm)
1	AHT with circular hole	73	1.007	1.1	6.9
2		93		1.8	7.5
3		113		1.3	6.85
4		133		1	6.8
5	AHT with square hole	73	1.01	1.8	4.5
6		93		2.1	4.7
7		113		2.83	4.25
8		133		2.45	4.7
9	AHT with multiple circular holes	73	1.01	1.66	4.85
10		93		1.32	4.25
11		113		1.73	4.6
12		133		0.95	3.8
13	AHT with perforation	73	1.014	1.1	4.56
14		93		0.8	4.75
15		113		1.05	3.4
16		133		1.2	4.78

## Data Availability

The data that support the findings of this study are available within the article.
